# Supplementary material for: Discovery of Flavonoids as Novel Inhibitors of ATP Citrate Lyase: Structure–Activity Relationship and Inhibition Profiles
Source: Int J Mol Sci. 2022 Sep 15;23(18):10747. doi: 10.3390/ijms231810747 (PMC9504748; doi:10.3390/ijms231810747)
Supplement: Supplementary file 1 [file ijms-23-10747-s001.zip › ijms-1893105-supplementary.pdf]

Discovery of flavonoids as novel inhibitors of ATP citrate lyase: structure-activity relationship and inhibition profile

Pan Wang[a,b], Tao Hou[a,c], Fangfang Xu[c], Fengbin Luo[c], Han Zhou[a,c], Fan Liu[a,b], Yanfang Liu[a,c], Jixia Wang[a,c,*], Zhimou Guo[a,c,*], Xinmiao Liang[a,c]

[a] CAS Key Laboratory of Separation Science for Analytical Chemistry, Dalian Institute of Chemical Physics, Chinese Academy of Sciences, Dalian 116023, China

[b] University of Chinese Academy of Sciences, Beijing 100000, China

[c] Jiangxi Provincial Key Laboratory for Pharmacodynamic Material Basis of Traditional Chinese Medicine, Ganjiang Chinese Medicine Innovation Center, Nanchang 330000, China

Corresponding Authors: Jixia Wang, Zhimou Guo

Tel.: +86 411 84379519; fax: +86 411 84379539.

E-mail addresses: jxwang@dicp.ac.cn, guozhimou@dicp.ac.cn

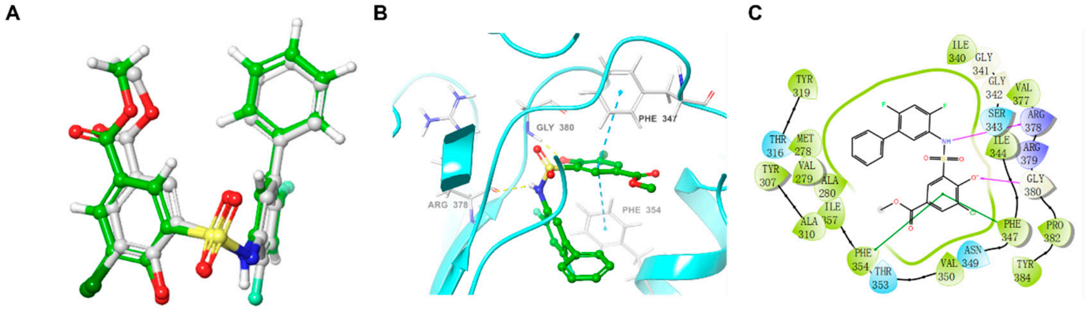

Figure S1. The re-docking of NDI-0911143 into prepared pocket. (A) Superposition of original and docked conformations of NDI-091143. (B) Three-dimensional presentation of NDI-091143 on ACLY. Carbon atoms in green, oxygen atoms in red, and hydrogen atoms in white; hydrogen-bond interactions were showed in dashed lines. (C) Two-dimensional presentation of NDI-091143 and ACLY. Hydrogen-bond interactions were marked with arrows.

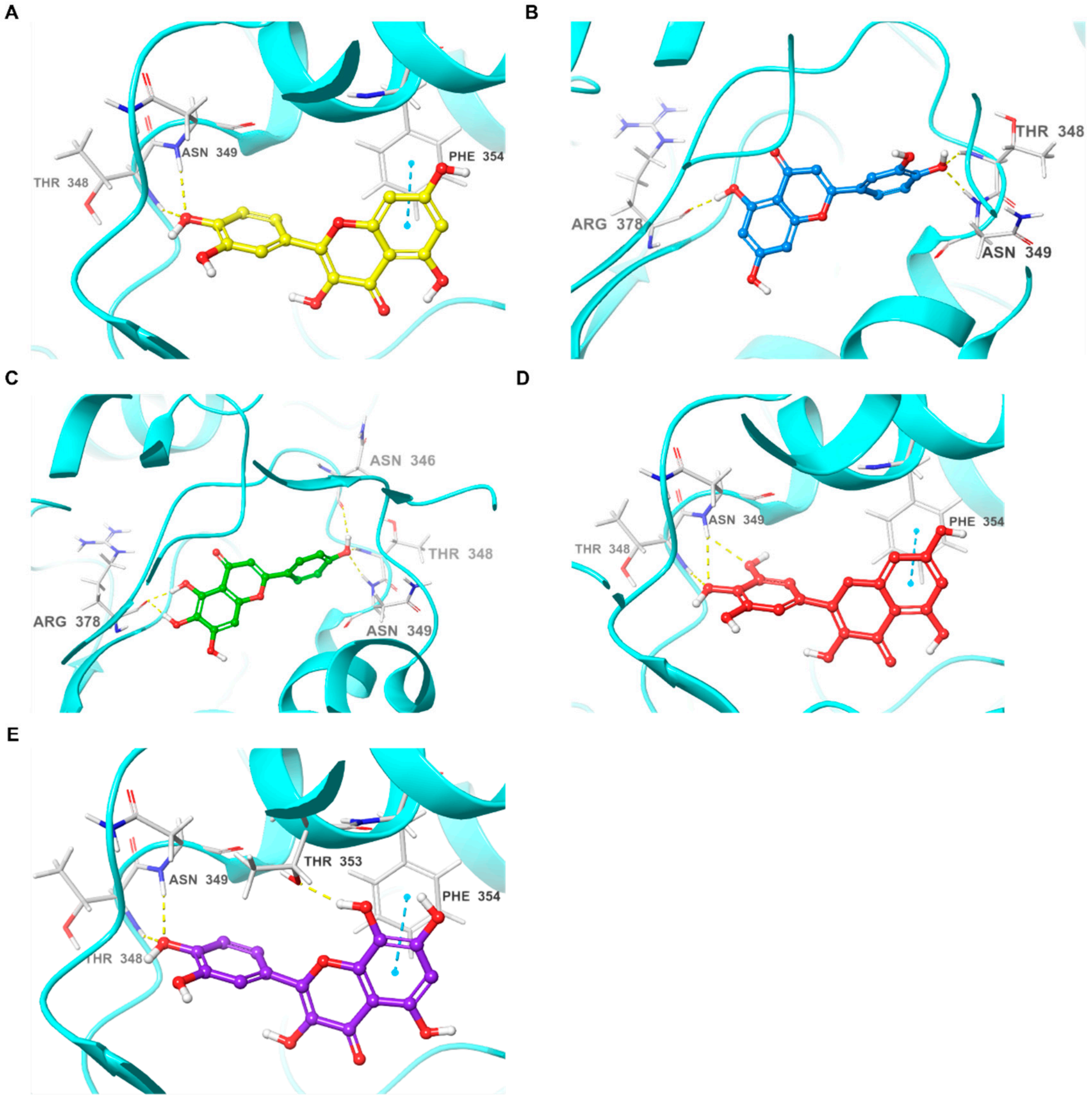

Figure S2. The docking studies of of the other ortho-dihydroxyphenyl flavonoids binding to ACLY. The interactions of ACLY with quercetin (A), luteolin (B), scutellarin (C), myricetin (D) and

gossypetin (E), respectively.

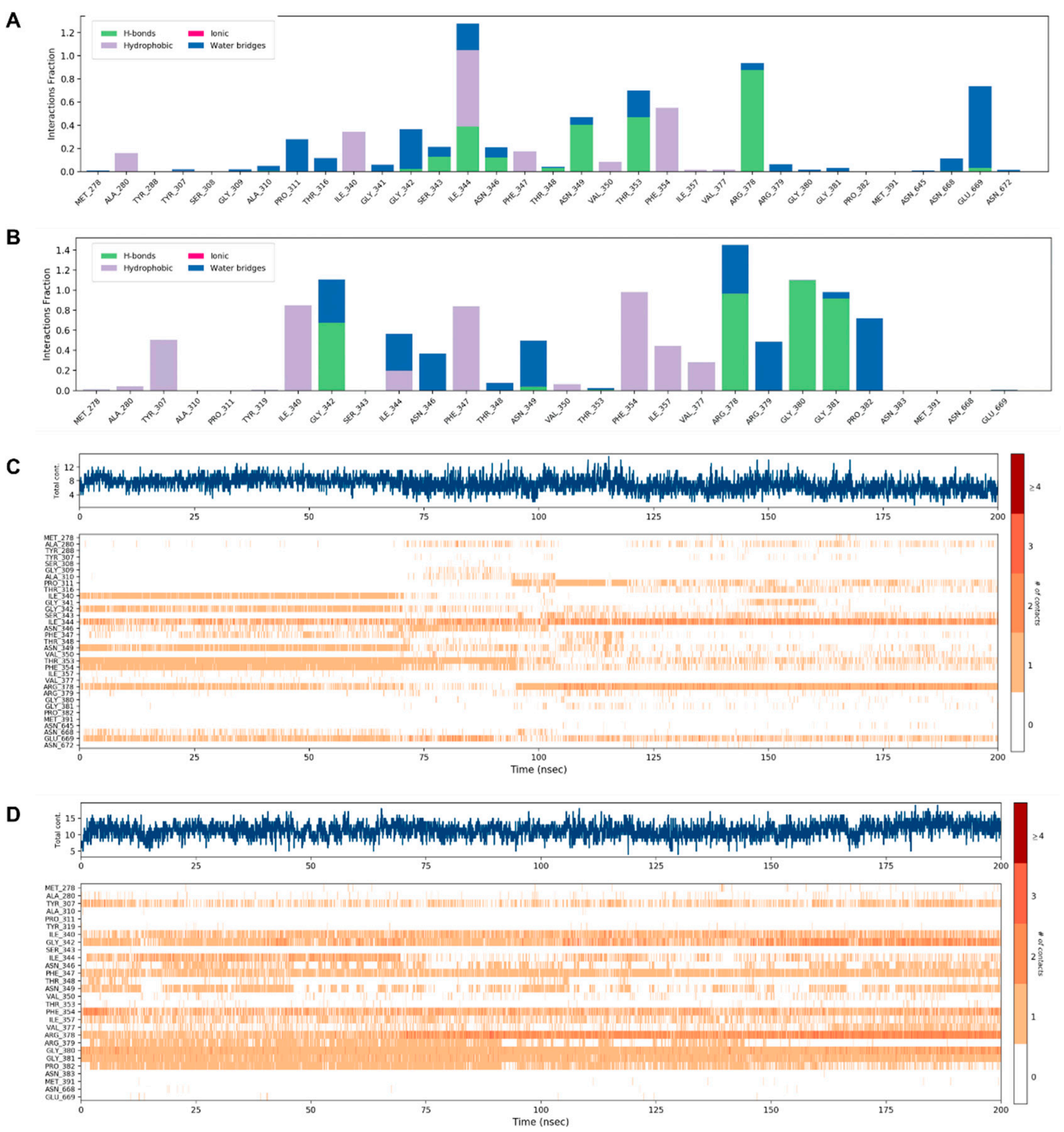

Figure S3. Interactions and contacts between ACLY and ligands throughout the simulation. (A) Interactions for herbacetin or NDI-091143 (B) binding to ACLY. (C) Contacts for herbacetin or NDI-091143 (D) binding to ACLY.

Table S1 Information of 138 selected flavonoids

	Compound	CAS	Formula
1	Narcissin	604-80-8	$C_{28}H_{32}O_{16}$
2	Kaempferitrin	482-38-2	$C_{27}H_{30}O_{14}$
3	Oroxylin A-7-O-glucuronide	36948-76-2	$C_{22}H_{20}O_{11}$
4	Apigenin-7-glucuronide	29741-09-1	$C_{21}H_{18}O_{11}$
5	Luteolin 7-O-glucuronide	29741-10-4	$C_{21}H_{18}O_{12}$
6	Hyperoside	482-36-0	$C_{21}H_{20}O_{12}$
7	Irisflorentin	41743-73-1	$C_{20}H_{18}O_{8}$

8	Tectoridin	611-40-5	$C_{22}H_{22}O_{11}$
9	Tectorigenin	548-77-6	$C_{16}H_{12}O_{6}$
10	Narirutin	14259-46-2	$C_{27}H_{32}O_{14}$
11	Poncirin	14941-08-3	$C_{28}H_{34}O_{14}$
12	Didymin	14259-47-3	$C_{28}H_{34}O_{14}$
13	Orientin	28608-75-5	$C_{21}H_{20}O_{11}$
14	Isoorientin	4261-42-1	$C_{21}H_{20}O_{11}$
15	Lonicerin	25694-72-8	$C_{27}H_{30}O_{15}$
16	Diosmetin-7-O-β-D-glucopyranoside	20126-59-4	$C_{22}H_{22}O_{11}$
17	Acacetin	480-44-4	$C_{16}H_{12}O_{5}$
18	Herbacetin	527-95-7	$C_{15}H_{10}O_{7}$
19	Taxifolin	480-18-2	$C_{15}H_{12}O_{7}$
20	Isoquercetin	482-35-9	$C_{21}H_{20}O_{12}$
21	Apigenin	520-36-5	$C_{15}H_{10}O_{5}$
22	Tricin	520-32-1	$C_{17}H_{14}O_{7}$
23	Hesperidin	520-26-3	$C_{28}H_{34}O_{15}$
24	Pinocembrin	480-39-7	$C_{15}H_{12}O_{4}$
25	Calycosin-7-O-β-D-glucoside	20633-67-4	$C_{22}H_{22}O_{10}$
26	Ononin	486-62-4	$C_{22}H_{22}O_{9}$
27	Calycosin	20575-57-9	$C_{16}H_{12}O_{5}$
28	Formononetin	485-72-3	$C_{16}H_{12}O_{4}$
29	Quercetin	117-39-5	$C_{15}H_{10}O_{7}$
30	Kaempferol	520-18-3	$C_{15}H_{10}O_{6}$
31	Naringenin	480-41-1	$C_{15}H_{12}O_{5}$
32	Rutin	153-18-4	$C_{27}H_{30}O_{16}$
33	Glabridin	59870-68-7	$C_{20}H_{20}O_{4}$
34	Liquiritigenin	578-86-9	$C_{15}H_{12}O_{4}$
35	Liquiritin	551-15-5	$C_{21}H_{22}O_{9}$
36	Liquiritin apioside	74639-14-8	$C_{26}H_{30}O_{13}$
37	Glabrone	60008-02-8	$C_{20}H_{16}O_{5}$
38	Licoflavone A	61153-77-3	$C_{20}H_{18}O_{4}$
39	Licoflavone B	91433-17-9	$C_{25}H_{26}O_{4}$
40	Licoflavone C	72357-31-4	$C_{20}H_{18}O_{5}$
41	Glabrol	59870-65-4	$C_{25}H_{28}O_{4}$
42	Genkwanin	437-64-9	$C_{16}H_{12}O_{5}$
43	Naringin	10236-47-2	$C_{27}H_{32}O_{14}$
44	Isorhamnetin	480-19-3	$C_{16}H_{12}O_{7}$
45	Baicalin	21967-41-9	$C_{21}H_{18}O_{11}$
46	Baicalein	491-67-8	$C_{15}H_{10}O_{5}$
47	Wogonin	632-85-9	$C_{16}H_{12}O_{5}$
48	Wogonoside	51059-44-0	$C_{22}H_{20}O_{11}$
49	Oroxylin A	480-11-5	$C_{16}H_{12}O_{5}$

50	Chrysin	480-40-0	$C_{15}H_{10}O_4$
51	Luteolin	491-70-3	$C_{15}H_{10}O_6$
52	Scutellarin	529-53-3	$C_{15}H_{10}O_6$
53	Scutellarin	27740-01-8	$C_{21}H_{18}O_{12}$
54	Neohesperidin	13241-33-3	$C_{28}H_{34}O_{15}$
55	Rhoifolin	17306-46-6	$C_{27}H_{30}O_{14}$
56	Hesperetin	520-33-2	$C_{16}H_{14}O_6$
57	Diosmetin	520-34-3	$C_{16}H_{12}O_6$
58	Sinensetin	2306-27-6	$C_{20}H_{20}O_7$
59	Nobiletin	478-01-3	$C_{21}H_{22}O_8$
60	5-O-Demethylnobiletin	2174-59-6	$C_{20}H_{20}O_8$
61	Tangeretin	481-53-8	$C_{20}H_{20}O_7$
62	Isosinensetin	17290-70-9	$C_{20}H_{20}O_7$
63	3,5,6,7,8,3',4'-Heptamethoxyflavone	1178-24-1	$C_{22}H_{24}O_9$
64	Kuwanon A	62949-77-3	$C_{25}H_{24}O_6$
65	Isoginkgetin	548-19-6	$C_{32}H_{22}O_{10}$
66	methylapigenin	5128-44-9	$C_{17}H_{14}O_5$
67	Icariin	489-32-7	$C_{33}H_{40}O_{15}$
68	Icaritin	118525-40-9	$C_{21}H_{20}O_6$
69	Anhydroicaritin	38226-86-7	$C_{21}H_{20}O_6$
70	Tricin	520-32-1	$C_{17}H_{14}O_7$
71	Chrysoeriol	491-71-4	$C_{16}H_{12}O_6$
72	Casticin	479-91-4	$C_{19}H_{18}O_8$
73	Catechin	154-23-4	$C_{15}H_{14}O_6$
74	Cyanidin chloride	528-58-5	$C_{15}H_{11}O_6$
75	Epicatechin	490-46-0	$C_{15}H_{14}O_6$
76	Eriodictyol	552-58-9	$C_{15}H_{12}O_6$
77	Eupatilin	22368-21-4	$C_{18}H_{16}O_7$
78	Galangin	548-83-4	$C_{15}H_{10}O_5$
79	Gardenin B	2798-20-1	$C_{19}H_{18}O_7$
80	Genistein	446-72-0	$C_{15}H_{10}O_5$
81	Glycitein	40957-83-3	$C_{16}H_{12}O_5$
82	Irigenin	548-76-5	$C_{18}H_{16}O_8$
83	Isoanhydroicaritin	28610-30-2	$C_{21}H_{20}O_6$
84	Isosilybin	72581-71-6	$C_{25}H_{22}O_{10}$
85	Kaempferide	491-54-3	$C_{16}H_{12}O_6$
86	Kushenol F	97938-30-2	$C_{25}H_{28}O_6$
87	Morin	480-16-0	$C_{15}H_{10}O_7$
88	Morusin	62596-29-6	$C_{25}H_{24}O_6$
89	Myricetin	529-44-2	$C_{15}H_{10}O_8$
90	Neobavaisoflavone	41060-15-5	$C_{20}H_{18}O_4$
91	Pectolinarigenin	520-12-7	$C_{17}H_{14}O_6$

92	7,4'-Dihydroxyflavone	2196-14-7	$C_{15}H_{10}O_4$
93	Gossypentin	489-35-0	$C_{15}H_{10}O_8$
94	Bilobtin	521-32-4	$C_{31}H_{20}O_{10}$
95	5,7-Dihydrox -4'-methoxyisoflavone	491-80-5	$C_{16}H_{12}O_5$
96	Daidzein	486-66-8	$C_{15}H_{10}O_4$
97	Daidzin	552-66-9	$C_{21}H_{20}O_9$
98	Dihydromyricetin	27200-12-0	$C_{15}H_{12}O_8$
99	Epmedin B	110623-73-9	$C_{38}H_{48}O_{19}$
100	Ginkgetin	481-46-9	$C_{32}H_{22}O_{10}$
101	Homoplantaginin	17680-84-1	$C_{22}H_{22}O_{11}$
102	Jaceosidin	18085-97-7	$C_{17}H_{14}O_7$
103	Procyanidin B1	20315-25-7	$C_{30}H_{26}O_{12}$
104	Procyanidin B2	29106-49-8	$C_{30}H_{26}O_{12}$
105	Puerarin	3681-99-0	$C_{21}H_{20}O_9$
106	7-(α-L-Rhamnopyranosyloxy)-2-(3,4-dihydroxyphenyl)-3,5-dihydroxy-4H-1-benzopyran-4-one	22007-72-3	$C_{21}H_{20}O_{11}$
107	Quercitrin	522-12-3	$C_{21}H_{20}O_{11}$
108	Sakuranetin	2957-21-3	$C_{16}H_{14}O_5$
109	Sciadopitysin	521-34-6	$C_{33}H_{24}O_{10}$
110	Silibinin	22888-70-6	$C_{25}H_{22}O_{10}$
111	Silicristin	33889-69-9	$C_{25}H_{22}O_{10}$
112	Silydianin	29782-68-1	$C_{25}H_{22}O_{10}$
113	Spinosin	72063-39-9	$C_{28}H_{32}O_{15}$
114	Vitexin	3681-93-4	$C_{21}H_{20}O_{10}$
115	5,7,3'-Trihydroxy-6,4',5'-triMethoxyflavone	78417-26-2	$C_{18}H_{16}O_8$
116	5,7,8,4''-Tetramethoxyflavone	6601-66-7	$C_{19}H_{18}O_6$
117	3'-Demethylnobiletin	112448-39-2	$C_{20}H_{20}O_8$
118	Podocarpusflavone A	22136-74-9	$C_{31}H_{20}O_{10}$
119	4'-O-Methylochnaflavone	49619-87-6	$C_{31}H_{20}O_{10}$
120	Baicalin methyl ester	82475-03-4	$C_{22}H_{20}O_{11}$
121	5-Methyl-7-methoxyisoflavone	82517-12-2	$C_{17}H_{14}O_3$
122	Isosakuranetin	480-43-3	$C_{16}H_{14}O_5$
123	Chrysosplenetin B	603-56-5	$C_{19}H_{18}O_8$
124	Isovitexin	29702-25-8	$C_{21}H_{20}O_{10}$
125	Typhaneoside	104472-68-6	$C_{34}H_{42}O_{20}$
126	(+)-Gallocatechin	970-73-0	$C_{15}H_{14}O_7$
127	Liquiritigenin	578-86-9	$C_{15}H_{12}O_4$
128	Naringenin	67604-48-2	$C_{15}H_{12}O_5$
129	(-)-Catechin hydrate	18829-70-4	$C_{15}H_{14}O_6$
130	Vitexin	3681-93-4	$C_{21}H_{20}O_{10}$
131	Vicenin	23666-13-9	$C_{27}H_{30}O_{15}$

132	Vicenin -1	35927-38-9	$C_{26}H_{28}O_{14}$
133	6-beta-D-Glucopyranosyl-8-beta-D-xylopyranosylapigenin	59914-91-9	$C_{26}H_{28}O_{14}$
134	Cynaroside	5373-11-5	$C_{21}H_{20}O_{11}$
135	Apigenin 7-glucoside	578-74-5	$C_{21}H_{20}O_{10}$
136	Hispidulin	1447-88-7	$C_{16}H_{12}O_6$
137	6-Methoxyluteolin	520-11-6	$C_{16}H_{12}O_7$
138	Plantagoside	78708-33-5	$C_{21}H_{22}O_{12}$

Table S2 Docking studies of ligands binding to ACLY

Ligand name	Docking score	MM-GBSA	Interaction
NDI-091143	-9.872	-57.26	PHE347|PHE354|ARG378|GLY380
Herbacetin	-9.673	-50.94	ASN346|THR348|ASN349|THR353
Quercetin	-7.092	-48.05	THR348|ASN349|PHE354
Luteolin	-8.000	-47.51	THR348|ASN349|ARG378
Scutellarin	-9.307	-49.06	ASN346|THR348|ASN349|ARG378
Myricetin	-7.064	-44.39	THR348|ASN349|PHE354
Gossypetin	-7.404	-52.31	THR348|ASN349|THR353|PHE354
